# Systemic Inflammatory Response and the Noble and Underwood (NUn) Score as Early Predictors of Anastomotic Leakage after Esophageal Reconstructive Surgery

**DOI:** 10.3390/jcm13030826

**Published:** 2024-01-31

**Authors:** Elke Van Daele, Hanne Vanommeslaeghe, Flo Decostere, Louise Beckers Perletti, Esther Beel, Yves Van Nieuwenhove, Wim Ceelen, Piet Pattyn

**Affiliations:** 1 Department of Gastrointestinal Surgery, Ghent University Hospital, C. Heymanslaan 10, B-9000 Ghent, Belgiumwim.ceelen@ugent.be (W.C.);; 2 Faculty of Medicine, Ghent University, C. Heymanslaan 10, B-9000 Ghent, Belgium; flo.decostere@ugent.be (F.D.); louise.beckers@ugent.be (L.B.P.); esther.beel@ugent.be (E.B.)

**Keywords:** NUn score, esophagectomy, anastomotic leakage, risk score, esophageal cancer, inflammatory biomarkers

## Abstract

Anastomotic leakage (AL) remains the main cause of post-esophagectomy morbidity and mortality. Early detection can avoid sepsis and reduce morbidity and mortality. This study evaluates the diagnostic accuracy of the Nun score and its components as early detectors of AL. This single-center observational cohort study included all esophagectomies from 2010 to 2020. C-reactive protein (CRP), albumin (Alb), and white cell count (WCC) were analyzed and NUn scores were calculated. The area under the curve statistic (AUC) was used to assess their predictive accuracy. A total of 74 of the 668 patients (11%) developed an AL. CRP and the NUn-score proved to be good diagnostic accuracy tests on postoperative day (POD) 2 (CRP AUC: 0.859; NUn score AUC: 0.869) and POD 4 (CRP AUC: 0.924; NUn score AUC: 0.948). A 182 mg/L CRP cut-off on POD 4 yielded a 87% sensitivity, 88% specificity, a negative predictive value (NPV) of 98%, and a positive predictive value (PPV) of 47.7%. A NUn score cut-off > 10 resulted in 92% sensitivity, 95% specificity, 99% NPV, and 68% PPV. Albumin and WCC have limited value in the detection of post-esophagectomy AL. Elevated CRP and a high NUn score on POD 4 provide high accuracy in predicting AL after esophageal cancer surgery. Their high negative predictive value allows to select patients who can safely proceed with enhanced recovery protocols.

## 1. Introduction

The incidence of esophageal cancer (EC) is increasing, making it the sixth leading cause of cancer-related mortality worldwide [1]. The prognosis remains poor with 5-year overall survival rates varying from 90% for stage I cancer to <10% for stage IV cancer patients. For locally advanced cancer, multimodality therapy followed by surgery has convincingly improved both local control and overall survival [2,3,4,5]. Surgical resection and lymphadenectomy remain crucial in the treatment of non-metastatic esophageal cancer patients [5,6]. However, the procedure is known for its potentially complicated postoperative course. Large benchmarking series report postoperative complications in more than 50% of cases, even in high-volume centers. Pulmonary complications (15–25%), cardiac events (14–15%), and the failure of the esophagogastric anastomosis (12–16%) remain the most important sources of both morbidity and mortality after esophagectomy [7,8,9]. Considerable improvements in surgical technique and perioperative care have resulted in 90-day mortality rates after esophagectomy of less than 5% in experienced centers [7,8,9]. However, the mortality of AL remains high, ranging from 7 to 17% [10,11]. The severity of AL depends on the location of the anastomosis, the estimated surface and circumference of the defect, the extent of contamination, the degree of sepsis, and the time from occurrence to diagnosis and therapy [12]. The early detection and management of an AL can prevent the development of mediastinitis-related sepsis and is critical to improving its outcome [13].

Inflammatory biomarkers have been previously proposed as easy and cheap tests for the early diagnosis of postoperative infectious complications after major surgery. C-reactive protein (CRP) is an acute-phase protein produced in response to infection, tissue damage, and ischemia. A low CRP on postoperative day (POD) 3 and 5 may rule out AL after esophagectomy [13]. However, it can be difficult to distinguish the normal systemic inflammatory response to surgical stress from AL-associated sepsis. Identifying a clinically relevant, easy-to-use scoring system may be helpful in the early diagnosis of AL, selecting patients for imaging, and tailoring AL management. Noble and Underwood introduced the NUn score, using the acute-phase markers white cell count (WCC), CRP, and albumin (Alb) as a predictor of AL and major postoperative complications [14]. The attempts to validate the score are limited and conflicting [15,16,17,18]. We aimed to determine the diagnostic accuracy of these inflammatory response biomarkers and the combined NUn score as early predictors of post-esophagectomy AL.

## 2. Materials and Methods

The study protocol was approved by the institutional review board of the Ghent University Hospital (reference: B670201111232).

### 2.1. Surgery and Postoperative Care

Transthoracic (sub)total esophagectomy with 2- or 3-field lymphadenectomy and a right intrathoracic (Ivor Lewis, IL or Transhiatal, THE) or cervical esophagogastric anastomosis (McKeown, McK) were performed. All procedures were performed by 2 surgeons (PP and EVD). The surgical approach included open as well as hybrid minimally invasive procedures (introduced in 2013). Fully minimal invasive esophagectomy (MIE) was introduced in 2014. All patients received an intrathoracic end-to-side or end-to-end circular esophagogastric anastomosis using a Premium Plus EEA™ (Medtronic, Dublin, Ireland) stapler (25 or 28 mm), or a standardized cervical end-to-side hand-sewn anastomosis. Patients recovered at the intensive care unit for 12–24 h and were then discharged to a dedicated gastrointestinal surgery ward. A nasogastric tube was kept in place during a period of 2–3 days. A water-soluble contrast swallow was obtained on the third postoperative day as a routine screening before initiating oral intake. Patients suspected of AL received an emergency CT scan with oral contrast and/or upper endoscopy. Anastomotic leakage was treated conservatively, endoscopically, or surgically, according to clinical presentation. Nutritional support was provided by a feeding jejunostomy. Since 2018, patients have been treated according to an Enhanced Recovery After Surgery (ERAS) protocol.

### 2.2. Patient Selection

This cohort study was based on data gathered from a prospective institutional database supplemented with data from the electronic patient records. Consecutive patients undergoing esophagectomy for cancer between January 2010 and December 2020, and fitting the criteria were included. Patients in whom the esophagus was replaced with a small bowel or colon, or who underwent concurrent laryngectomy, were excluded. 

### 2.3. Outcomes

Individual collected data included demographics, American Society of Anesthesiologists (ASA) score, tumor characteristics, the type of neoadjuvant therapy, surgical details, pathology reports, laboratory results, and postoperative morbidity and mortality until 90 days postoperatively. Pathological staging was based on the 7th AJCC TNM classification manual. Postoperative morbidity and mortality were classified using the European Complication Consensus Group (ECCG) platform [19] and graded according to the Clavien Dindo classification [20]. Anastomotic leakage was defined as a full thickness gastrointestinal defect involving the esophagus, anastomosis, staple line, or conduit, irrespective of presentation or method of identification, according to the ECCG classification. Results are reported according to the “Strengthening the Reporting of Observational Studies in Epidemiology” (STROBE) guidelines [21].

### 2.4. Inflammatory Biomarkers and the NUn Score

Acute-phase markers were retrieved from the daily blood samples postoperatively. WCC was measured in cells ×10^3^/µL (reference range 3.6–9.3 10^3^/µL) and converted to 10^9^/L for the NUn score calculation. Serum concentrations of albumin were expressed in g/L (normal range 35–52 g/L) and CRP in mg/L (normal range < 0.5 mg/L). The NUn score was calculated according the original Noble formula: 11.3894 + (0.005 × CRP in mg/L) + (0.186 × WCC in 10^9^/L) − (0.174 × albumin in g/L). Missing data were replaced using the last observation carried forward approach.

### 2.5. Statistical Analysis

All analyses were performed using IBM SPSS^®^ version 28 for Windows^®^ and Sigmaplot^®^ version 13 for Windows^®^. Continuous data are summarized as means with standard deviations (SD), or as medians with interquartile ranges (IQR). Categorical data are reported using frequencies and percentages. Independent samples *t* test, Pearson chi square, Fisher’s exact, and Mann–Whitney U tests were used to compare means and proportions. The significance of the different covariates in the prediction of AL was assessed using univariate analysis. The predictive accuracy of the biomarkers and the NUn score was assessed using receiver operating curve (ROC) analyses and the area under the curve (AUC). Sensitivity, specificity, and positive and negative predictive value were calculated for the determined cut-off values of the biomarkers and the NUn score.

## 3. Results

### 3.1. Demographics of the Study Cohort

Between January 2010 and December 2020, 668 esophagectomy patients were identified matching the inclusion criteria. Demographic data and their univariable association with AL are detailed in Table 1. The mean age was 64.0 ± 12.2 years (78.9% male). Overall, 74 patients (11.1%) experienced an AL. The majority of patients were treated for an adenocarcinoma (67.5%). Univariable analysis could not identify statistically significant differences in demographics, comorbidities, neoadjuvant treatment regimens, histology, or clinical staging between the patients with and without leakage, except for a higher percent of ASA 3 patients in the AL group. The surgical procedure, approach, and conditions did, however, significantly influence the AL rate, with a significantly higher AL rate in patients with a cervical anastomosis (McK 28% vs. IL 10.9% vs. THE 5.2%, *p* = 0.010), after total minimally invasive surgery (16.8% vs. 8.6% after both open and hybrid procedures, *p* = 0.008), and when an emergency procedure was performed. AL was defined according to the ECCG guidelines, diagnosed on CT-scan and/or upper GI endoscopy, and graded according to both the CD (17.6% gr 2; 1.4% gr 3a; 43.2% gr 3b; 27% gr 4a; 8.1% gr 4b; and 2.7% gr 5) and ECCG grading system (18.9% type 1; 12.2% type 2; and 68.9% type 3).

### 3.2. Mean Levels of Inflammatory Biomarkers and Severity of the AL

**Figure 1:**
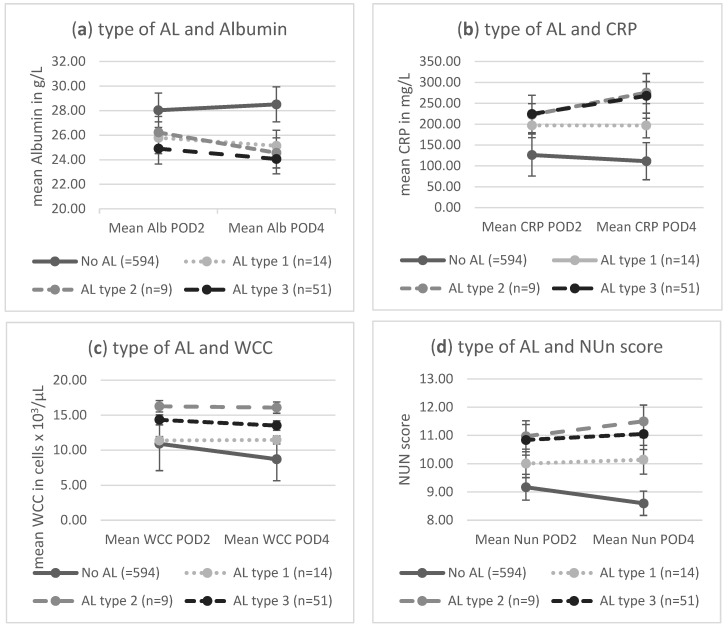
Mean levels of albumin, CRP, WCC, and NUn score on postoperative day 2 and 4 in patients with and without AL, stratified by ECCG type of AL (data displayed as means with standard deviation). (**a**) Correlation between mean Alb and type of AL, (**b**) correlation between mean CRP and type of AL, (**c**) correlation between mean WCC and type of AL, and (**d**) correlation between mean NUn and type of AL.

CRP was available in 642 patients on POD 2 and 613 patients on POD 4. WCC was measured in 645 patients on POD 2 and 662 patients on POD 4. Albumin was available for 596 patients on POD 2 and 615 on POD 4. Missing data were replaced using the last observation carried forward approach. NUn scores were calculated for all but five patients on POD 2 and four patients on POD 4. Mean CRP, WCC, and combined NUn scores were significantly higher in AL patients compared to the non-AL patients, and this significance was confirmed for all ECCG AL types. Mean Alb was significantly lower in the AL group. Mean CRP and WCC levels were higher in patients with a more sever ECCG AL grade, specifically when type 1 leaks were compared to type 2 and type 3 leaks. The significance was present for the evaluated biomarkers both on POD 2 and POD 4 (Figure 1). All biomarkers were identified as significant markers for AL on univariate analysis (Table 2). 

### 3.3. Optimal Cut-Off and Predictive Accuracy of Albumin

**Figure 2:**
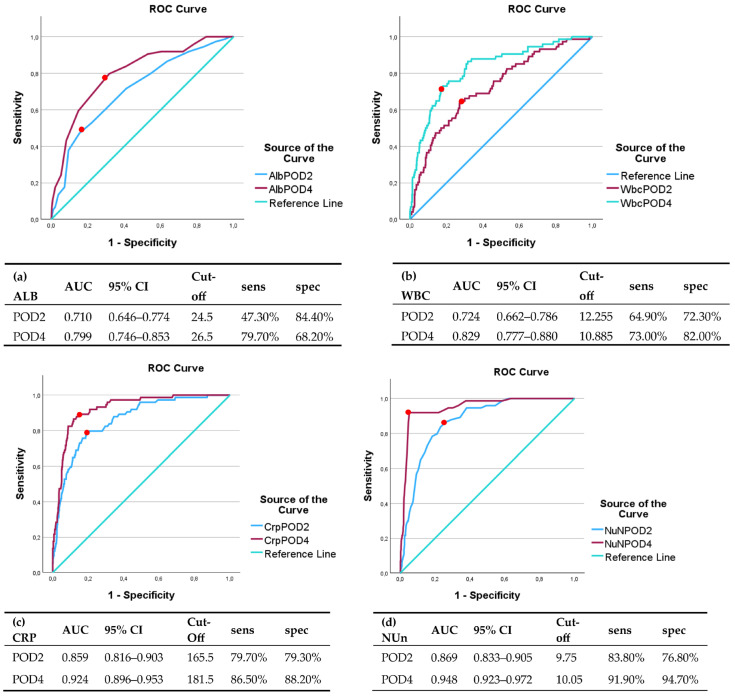
Receiver operating curve (ROC) for albumin, C-reactive protein, white cell count, and the NUn score on POD 2 (light blue) and POD 4 (dark red) and their diagnostic accuracy in detecting AL. (**a**) ROC curve for Alb, (**b**) ROC curve for WCC, (**c**) ROC curve for CRP, and (**d**) ROC curve for the NUn score. The *X* axis resembles the true positive rate (=sensitivity) and the *Y* axis resembles the false positive rate (=1-specificity). The red dot is the cut-off value, and Youden’s J statistic is used to select the optimal predicted probability cut-off. It is the maximum vertical distance between the ROC curve and the diagonal line.

Mean albumin levels for patients with and without AL were 25.2 versus 28.0 g/L on POD 2 (*p* < 0.001) and 24.3 versus 28.5 g/L on POD 4 (*p* < 0.001). Figure 2 shows the ROC curve analyses of albumin, with a fair performance on POD 2 (AUC 0.710, 95% CI: 0.646–0.774) and POD 4 (AUC 0.799, 95% CI: 0.746–0.853). A POD 4 albumin threshold of 26.5 g/L had the highest, but still limited diagnostic accuracy, with a sensitivity of 80% and a specificity of 68%.

### 3.4. Optimal Cut-Off and Predictive Accuracy of CRP

The mean CRP levels for patients with and without AL were 218.9 versus 125.9 mg/L, respectively, on POD 2 (*p* < 0.001) and 255.2 mg/L versus 111.1 on POD 4 (*p* < 0.001). Figure 2 shows the ROC curve analyses of CRP, with a good performance on POD 2 (AUC 0.859, 95% CI: 0.816–0.903) and an excellent performance on POD 4 (AUC 0.924, 95% CI: 0.896–0.953). A POD 4 CRP threshold of 181.5 mg/L had the highest diagnostic accuracy compared to all the other individual markers. This resulted in a sensitivity of 87%, a specificity of 88%, a negative predictive value of 98%, and a positive predictive value of 48%. 

### 3.5. Optimal Cut-Off and Predictive Accuracy of WCC

Mean WCC levels were significantly higher for patients with AL (14.0 and 13.4 × 10^3^/µL on POD 2 and 4) compared to the those for patients without an AL (10.9 and 8.7 × 10^3^/µL on POD 2 and 4) (*p* < 0.001). Figure 2 shows the ROC curve analyses of WCC, with a fair performance on POD 2 (AUC 0.724 95% CI: 0.662–0.786) but a good performance on POD 4 (AUC 0.829, 95% CI: 0.777–0.880). A POD 4 WCC cut-off of 10.9 × 10^3^/µL resulted in a sensitivity of 73%, a specificity of 82%, a negative predictive value of 96%, and a positive predictive value of 33%. 

### 3.6. Optimal Cut-Off and Predictive Accuracy of the NUn Score

Patients with AL presented a mean NUn score of 10.7 on POD 2 and 10.9 on POD 4, compared to a 9.2 score on POD 2 and 8.6 score on POD 4 in the non-AL group (*p* < 0.001). The presence of a NUn score > 10 on POD4, as presented by Noble and Underwood, was identified as a significant risk factor for AL both in the univariate and multivariate analysis in this study group. Figure 2 shows the ROC curve analyses of the NUn score, with a good performance on POD 2 (AUC 0.869, 95% CI: 0.833–0.905) and an excellent performance on POD 4 (AUC 0.948, 95% CI: 0.923–0.972). A POD 4 NUn score of >10 had the highest diagnostic accuracy compared to all the other individual markers, with a sensitivity of 92%, a specificity of 95%, a negative predictive value of 99%, and a positive predictive value of 68% (Table 3).

## 4. Discussion

The failure of the esophagogastric anastomosis (12–16%) remains the most important source of prolonged hospital stay, increased risk for reoperation, stenosis, short-term reduced quality of live, increased costs, and increased perioperative death [12,22]. The effect of post-esophagectomy AL on long term oncological and functional outcome is still under debate [23,24,25,26]. The clinical presentation of AL is diverse and its severity ranges widely, mainly determined by the location and extent of the defect, the presence of contamination and sepsis, and the time from onset to treatment [12]. Early diagnosis and treatment helps to prevent subsequent sepsis and improves AL-related outcomes. This observational study demonstrates the clinical utility of both CRP and the NUn score in postoperative AL monitoring in esophagectomy patients. The high NPV and the rather low PPV, however, suggest that their main value is not the early detection, but rather the exclusion of an AL.

A postoperative drop in **Albumin** (Alb) is thought to be a marker for surgical stress. The low concentrations of Alb and prealbumin on POD 4–6 are identified as potential risk factors for AL. Five studies evaluated postoperative Alb in relation to AL but only Noble reported a significant association with a POD 5 cut-off < 22.5 g/L with fair performance (AUC 0.742) [14,15,16,27,28,29,30]. Our analyses identified an equally fair performance for Alb with threshold values of <24.5 on POD 2 (AUC 0.710) and <26.5 g/L on POD 4 (AUC 0.799). Given its limited accuracy, the authors do not advocate Alb alone as a predictive marker of AL. However, pre albumin, Alb in combined scores (e.g., Alb/CRP ratio, CART algorithm), and a perioperative Alb decrease of 11 g/L seem more promising as predictive markers [28,30,31]. 

Elevated **CRP** levels are the most commonly identified markers for post-esophagectomy complications [14,15,16,17,18,28,29,30,31,32,33,34,35,36,37,38,39,40,41,42,43,44,45,46,47]. CRP is an acute-phase protein synthesized in the liver in response to endotoxins, and its levels commonly increase within 6 h after the onset of the inflammation. It is a marker for acute inflammation with a high sensitivity but often low specificity for its inflammatory origin. CRP values have been studied from POD 1 to 10, with most studies focusing on POD 3–5. However, the earlier the AL is suspected, the better. We therefore focused on POD 2–4, as POD1 CRP showed low diagnostic performance in previous studies. In this study, the mean CRP levels on POD 2–4 were significantly higher in the AL group and proportionally correlated to the ECCG type of the AL, a finding consistent with Hagens et al.’s observations; however, due to the small sample size in that cohort, they could not prove statistical significance [47]. ROC curves were plotted to identify a CRP cut-off level of 165 mg/L on POD 2 with good diagnostic performance (AUC 0.859) and a cut-off level of 181 mg/L on POD 4 with excellent performance (AUC 0.924). Six other studies evaluated POD 2 CRP with varying thresholds from 177 to 300 mg/L [14,34,36,40,41,42]. All studies identified higher thresholds than ours on POD 2, and with lower AUCs, except Ji who identified a cut-off of 177 mg/L on POD 2 with a good performance (AUC 0.994, sens 90%, and spec 95%) similar to this study. Our POD 4 cut-off of 181 mg/L was significantly higher than the cut-off level of 111 mg/L reported by Miki [44] and 106 mg/L by Stuart [45], probably because they only included MIE patients. But it was in line with the threshold value of 177 mg/L published in a meta-analysis by Aiolfi who included all types of esophagectomy [13]. Based on the high AUC, the relevant sensitivity, specificity, low PPV, but high NPV, we could identify POD 2–4 CRP levels only to be useful in the exclusion and not in the diagnosis of an AL. This is consistent with most other studies that identify CRP as a negative predictor for AL.

Mean **WCC** levels were significantly different between the AL and the non-AL patients. However, our study identified WCC on POD 2 to have only a fair diagnostic accuracy (AUC 0.724) while in POD 4 it had a good diagnostic performance (AUC 0.829). The high NPV and low PPV again suggest clinical use as negative predictor instead of a diagnostic tool. Multiple studies evaluated WCC but only three reported cut-off values; however, they did so only on POD 3 and 5 and with poor diagnostic accuracy, eliminating the possibility for comparison [14,15,16,18,27,32,33,34,44,48].

Noble combined CRP, Alb, and white cell count in the **NUn score**, in an attempt to increase their accuracy as a AL predictor [14]. Findlay and Paireder failed to validate the score, potentially because they included all AL types, both symptomatic and asymptomatic, compared to Noble who included only “leaks sufficient to cause symptoms” [15,17]. Bundred, however, successfully validated the score’s cut-off value of 10 on POD 4, with a fair diagnostic accuracy (AUC 0.77) and including only symptomatic leaks, confirmed by radiology or endoscopy consistent to Noble’s definition [16]. Liesenfeld identified a sign difference between the mean NUn score of AL negative and positive patients (8.6 vs. 9.1, *p* = 0.006), but the optimal cut-off value recommended by Noble could not be confirmed as an AL predictor [18]. In this study, the NUn score seemed to have the highest accuracy of all tested biomarkers, and not just for the symptomatic AL patients as initially proven by Noble and validated by Bundred, but in all ECCG types of AL (whereas Findlay and Paireder failed to validate the score in a similar cohort).The presence of a NUn score > 10 on POD 4 was identified as a significant risk factor for AL, and the ROC curve analysis showed good performance on POD 2 (AUC 0.869) and an excellent performance on POD 4 (AUC 0.948), validating the score in this cohort.

This study has multiple pitfalls, as it is retrospective in nature, but based on prospectively collected data. We analyzed a heterogenic esophagectomy population including different procedures, approaches, and types of surgery, all known to have an impact on the AL rate, potentially biasing the results. However, we wanted to evaluate cheap and easily available tests and standardize their clinical use in postoperative monitoring for all esophagectomy patients. EC cancer is a rare disease resulting in a limited amount of annual esophagectomies. Nevertheless, we present a large population for a single-center observational study. Moreover, this is the first study to validate the NUn-score for all ECCG types in AL.

## 5. Conclusions

CRP and the NUn score both show good diagnostic performance on POD 2 and excellent performance on POD 4. They are, however, only valuable for AL exclusion, which can be useful in algorithms for a safe and early discharge. There is no single non-invasive test that can rule out AL, but patients with a CRP < 165 mg/L on POD 2 can proceed with oral intake according to the local ERAS protocol, and patients with a CRP < 181 mg/L or a NUn score < 10 on POD 4 are unlikely to develop an AL and can safely be discharged when clinically possible.

While highly elevated CRP levels have been consistently associated with post-operative inflammation and inflammatory complications, it is essential to acknowledge that they should not be used in isolation. CRP and NUn score kinetics over time may provide additional insights into the severity and the progression of a post-esophagectomy complications. However, daily CRP monitoring in the postoperative follow-up of esophagectomy patients seems to be a valuable strategy for the early detection of AL. Its negative predictive value and dynamic response make it a useful tool. However, clinical assessment, imaging studies, and endoscopic evaluations should be considered in junction with CRP. Based on our results, we created a center-specific diagnostic algorithm including clinical signs, CRP, NUn score, drain amylase, chest CT scan, and upper GI endoscopy to facilitate early diagnostic and surgical decision making for patients suspected for AL.

## Figures and Tables

**Table 1:** Baseline characteristics.

		All Patients (*n* = 668)	No AL (*n* = 594)	AL (*n* = 74)	*p* Value
Age, (y)	Mean ± SD	64.0 ± 12.2	64.8 ± 10.2	65.6 ± 8.9	0.508
BMI (kg/m^2^)	Mean ± SD	25.3 ± 4.6	25.2 ± 4.5	25.9 ± 4.9	0.252
ASA score, *n* (%)	1	27 (4.0%)	24 (4.1%)	3 (4.1%)	**0.036**
	2	286 (42.8%)	261 (43.9%)	25 (33.7%)	
	3	335 (50.1%)	292 (49.2%)	43 (58.1%)	
	4	4 (0.6%)	2 (0.3%)	2 (2.7%)	
Gender, *n* (%)	Male	527 (78.9%)	468 (78.8%)	59 (79.7%)	0.851
	Female	141 (21.1%)	126 (21.2%)	15 (20.3%)	
Comorbidities, *n* (%)	Kidney disease	21 (3.1%)	19 (3.2%)	2 (2.7%)	0.818
	Cardiovascular disease	257 (38.5%)	226 (38.0%)	31 (41.9%)	0.522
	Pulmonary disease	161 (24.1%)	140 (23.6%)	21 (28.4%)	0.362
	Diabetes	88 (13.2%)	75 (12.6%)	13 (17.6%)	0.236
	Smoking	230 (34.4%)	200 (33.7%)	30 (40.5%)	0.241
	Corticosteroids	20 (3.0%)	16 (2.7%)	4 (5.4%)	0.197
Tumor Location, *n* (%)	Proximal	17 (2.5%)	12 (2.0%)	5 (6.8%)	0.094
	Mid	121 (18.1%)	110 (18.5%)	11 (14.9%)	
	Distal	402 (60.2%)	357 (60.1%)	45 (60.8%)	
	GEJ	128 (19.2%)	115 (19.4%)	13 (17.6%)	
Neoadjuvant therapy, *n* (%)	None	179 (26.8%)	158 (26.6%)	21 (28.4%)	0.932
	Chemotherapy	97 (14.5%)	87 (14.6%)	10 (13.5%)	
	Radiochemotherapy	392 (58.7%)	349 (58.8%)	43 (58.1%)	
Histology, *n* (%)	Adeno Ca	451 (67.5%)	402 (67.7%)	49 (66.2%)	0.719
	Squamous cell Ca	200 (29.9%)	176 (29.6%)	24 (32.4%)	
	Other	17 (2.5%)	16 (2.7%)	1 (1.4%)	
cT-stage, *n* (%) *	Tx	8 (1.2%)	7 (1.2%)	1 (1.4%)	0.641
	T1	56 (8.4%)	49 (8.2%)	7 (9.5%)	
	T2	136 (20.4%)	118 (19.9%)	18 (24.3%)	
	T3	455 (68.1%)	407 (68.5%)	48 (64.9%)	
	T4	13 (1.9%)	13 (2.2%)	0 (0.0%)	
cN-stage, *n* (%) *	N0	227 (34.0%)	203 (34.2%)	24 (32.4%)	0.898
	N1	308 (46.1%)	276 (46.5%)	32 (43.2%)	
	N2	112 (16.8%)	97 (16.3%)	15 (20.3%)	
	N3	13 (1.9%)	11 (1.9%)	2 (2.7%)	
cM-stage, *n* (%) *	M0	625 (93.6%)	556 (93.6%)	69 (93.2%)	0.989
	M1	35 (5.2%)	31 (5.2%)	4 (5.4%)	
Procedure, *n* (%)	IL	586 (87.7%)	522 (87.9%)	64 (86.5%)	**0.010**
	McK	25 (3.7%)	18 (3.0%)	7 (9.5%)	
	THE	57 (8.5%)	54 (9.1%)	3 (4.1%)	
Approach, *n* (%)	Open	327 (49.0%)	299 (50.3%)	28 (37.8%)	**0.008**
	Hybride	139 (20.8%)	127 (21.4%)	12 (16.2%)	
	MIE	202 (30.2%)	168 (28.3%)	34 (45.9%)	
Type of surgery, *n* (%)	Elective	608 (91.0%)	545 (91.8%)	63 (85.1%)	**<0.001**
	Emergency	5 (0.7%)	1 (0.2%)	4 (5.4%)	
	Salvage	55 (8.2%)	48 (8.1%)	7 (9.5%)	

SD, standard deviation; BMI, body mass index; ASA, American Society of Anesthesiologists; GEJ, gastro esophageal junction; IL, Ivor Lewis; McK, McKeown; THE, transhiatal esophagectomy; * cTNM staging according to the AJCC 8th edition. Bold values state statistical significance.

**Table 2:** Univariate analysis of the mean biomarkers and NUn score on POD 2 and 4 according to the ECCG type of AL.

	No AL (=594)	AL Type 1 (*n* = 14)	AL Type 2 (*n* = 9)	AL Type 3 (*n* = 51)	*p* Value
Alb POD 2 (mean ± SD)	28.0 (±3.7)	25.8 (±2.6)	26.2 (±4.4)	24.9 (±4.1)	<0.001
Alb POD 4 (mean ± SD)	28.5 (±3.9)	25.1 (±3.4)	24.6 (±4.0)	24.1 (±3.2)	<0.001
CRP POD 2 (mean ± SD)	125.9 (±55.4)	197.0 (±89.8)	222.3 (±73.2)	224.4 (±67.9)	<0.001
CRP POD 4 (mean ± SD)	111.1 (±62.8)	196.8 (±84.9)	275.4 (±86.4)	267.6 (±74.0)	<0.001
WCC POD 2 (mean ± SD)	10.9 (±5.2)	11.4 (±3.6)	16.3 (±6.5)	14.3 (±4.2)	<0.001
WCC POD 4 (mean ± SD)	8.7 (±2.9)	11.46 (±3.7)	16.1 (±5.7)	13.5 (±4.5)	<0.001
NUn POD 2 (mean ± SD)	9.2 (±1.2)	10.0 (±0.8)	10.9 (±1.4)	10.8 (±1.0)	<0.001
NUn POD 4 (mean ± SD)	8.6 (±1.0)	10.1 (±1.2)	11.5 (±0.8)	11.1 (±0.9)	<0.001

**Table 3:** Threshold values for Alb, CRP, WCC, and the NUn score and their diagnostic accuracy for AL.

Variable		AUC	95% CI	*p* Value	Cut-Off	Sens	Spec	PPV	NPV	PLR	NLR
Alb	POD2	0.710	0.646–0.774	<0.001	24.5	47.30%	84.40%	27.42%	92.78%	3.032	0.624
	POD4	0.799	0.746–0.853	<0.001	26.5	79.70%	68.20%	23.79%	96.42%	2.506	0.298
CRP	POD2	0.859	0.816–0.903	<0.001	165.5	79.70%	79.30%	32.42%	96.90%	3.850	0.256
	POD4	0.924	0.896–0.953	<0.001	181.5	86.50%	88.20%	47.73%	98.13%	7.330	0.153
WCC	POD2	0.724	0.662–0.786	<0.001	12.255	64.90%	72.30%	22.59%	94.30%	2.343	0.486
	POD4	0.829	0.777–0.880	<0.001	10.885	73.00%	82.00%	33.57%	96.06%	4.056	0.329
NUn	POD2	0.869	0.833–0.905	<0.001	9.75	83.80%	76.80%	31.03%	97.44%	3.612	0.211
	POD4	0.948	0.923–0.972	<0.001	10.05	91.90%	94.70%	68.36%	98.95%	17.340	0.086

AUC, area under the curve; CI, confidence interval; Sens, sensitivity; Spec, specificity; PPV, positive predictive value; NPV, negative predictive value; PLR, positive likelihood ratio; NLR, negative likelihood ratio.

## Data Availability

All relevant data are available within the paper. Additional data if needed can be obtained from the corresponding author after approval by the local ethical committee.
